# A smart mask to enforce social contracts based on IOTA Tangle

**DOI:** 10.1371/journal.pone.0292850

**Published:** 2024-03-22

**Authors:** Lianna Zhao, Pietro Ferraro, Robert Shorten

**Affiliations:** Dyson School of Design Engineering, Imperial College London, London, United Kingdom; Università di Pisa, ITALY

## Abstract

In this paper we present the design for a smart-mask to mitigate the impact of an airborne virus such as COVID-19. The design utilises recent results from feedback control theory over a distributed ledger that have been developed to enforce compliance in a pseudo-anonymous manner. The design is based on the use of the IOTA distributed ledger. A hardware-in-the-loop simulation based on indoor positioning, paired with Monte-Carlo simulations, is developed to demonstrate the efficacy of the designed prototype.

## 1 Introduction

During the COVID pandemic, mask wearing played a significant role in eventually controlling the spread of the virus [1]. According to European Centre for Disease Prevention and Control, from 31 December 2019 to 17 March 2022, 458,179,120 cases of COVID-19 (under the applied case definitions and testing strategies in the affected countries) have been reported, including 6,058,022 deaths; see for example, https://www.ecdc.europa.eu/en/geographical-distribution-2019-ncov-cases. If not properly controlled, the virus might once again emerge and spread across the population, leading to high mortality rates and hospitalisations. Even as the amount of infected people abates, the need to wear face-masks remains in many aspects of daily life in some countries; for example, in passenger planes, buses, and trains. In such situations, enforcement of mask wearing is the responsibility of *observers*, such as flight attendants, rather than the mask wearer. Often, this leads to situations where either compliance is not enforced, or where an unreasonable burden is placed on these *observers*. In this paper, we explore ways to encourage people to wear masks properly, especially in confined and crowded spaces (for example: airplanes). Importantly, we wish to design mechanisms where compliance with mask wearing (or more general social contracts) remains with the mask wearer, rather than with *observers*. To do this we build on our previous work done in [4], in which the authors discuss a general framework, based on control theory, to regulate compliance to social contracts in the sharing economy domain.

**Fig 1:**
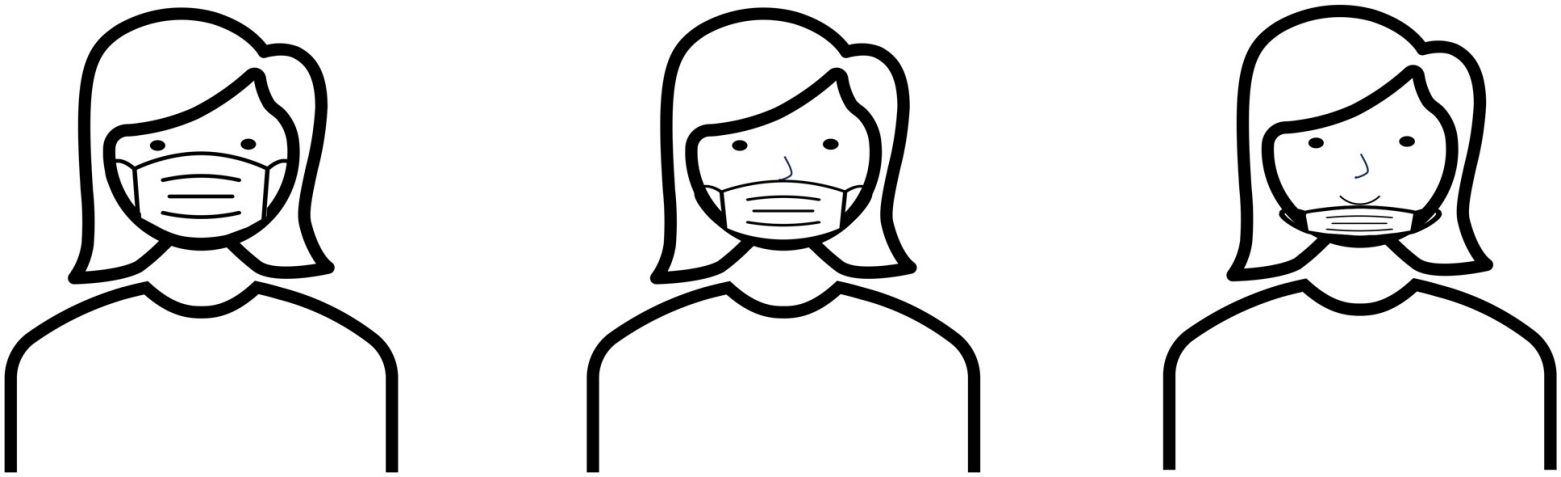
Correct and incorrect mask wearing.

The objective of this paper is to present a Proof-of-Concept (PoC) smart mask that can detect people’s mask-wearing status, and then to incentivise people’s behaviour with a bonding mechanism to wear masks efficiently in confined and crowded spaces. By wearing masks correctly, we mean that people use a mask to cover both their mouth and nose at the same time as depicted by the person on the left in Fig 1. In contrast, the middle and right individuals in the same figure are illustrations of not proper mask wearing.

Our work also builds on the idea of a personalised dynamic pricing strategy to assist in ensuring that agents in a population comply with a social contract. Here, by a social contract, we mean a set of guidelines that must be followed to ensure the proper utilisation of a resource or object. Such ideas are common in the sharing economy where goods should be returned on time and in good order, and in the circular economy where sorting of rubbish is incentivised using bonds (deposits) to encourage that consumers sort certain types of household waste. In our case, the agreement of wearing a mask correctly, in the context of COVID-19, is a social contract. While there are other papers on this and related topics-for example-see [2, 3], our work is based on a novel perspective on this problem that is presented in [4]. In [4], the authors propose a personalised feedback control based on a DLT system that encourages compliance with a social contract. The authors in this paper also provide a theoretical analysis of the designed system’s convergence. In the context of [4], the role of the DLT is to provide a more secure and privacy-aware structure than in centralised alternatives, and to provide a platform for creating a personalised economic commitment algorithm. For the convenience of remaining discussion in the present paper, we refer to the algorithm in [4] as Personalised Feedback Control Algorithm (PFCA). We note also that the idea of incentives is also being explored more widely in the circular economy and that similar ideas have appeared in various applications and prototypes [5, 6], such as Kupcrush (https://biotasphere.com/showcase/). Kupcrush uses DLTs to make cups into economic agents through the use of Digital Twins. Each cup is associated with a digital identity and a wallet. The aim is to incentivise the consumer who is using the cup to recycle it correctly by rewarding every actor in the circular economy with a micro-reward as the cup moves through the recycling chain until it is ultimately recycled into a new cup. Finally, we note that the issue of compliance has been discussed in other application domains [7, 8]. However, there are significant limitations in these works. For example, the systems advocated in these papers are often centralised and lack anonymity and other privacy guarantees. Accordingly, the main contributions of this paper are the following.

- First, we propose a digital bonding system, based on the IOTA Tangle technology and using ideas from feedback theory, to encourage adherence of a population to a social contract; in this case wearing a smart mask [9–11].
- Second, we present a smart mask design. The smart mask is designed to detect people’s mask wearing status. A prototype based on a Raspberry Pi 3B [12] hardware platform is described, complete with integration of the aforementioned digital bonding system.
- Agent-based simulations, as well as hardware-in-the-loop simulations are presented to illustrate the efficacy of the proposed system.

In what follows we discuss the design of the aforementioned system. Before proceeding we make the following important comments.

A. Our smart mask system is designed to operate in sensitive situations; for example, in a passenger aircraft during a pandemic such as COVID-19. During COVID-19 it was frequently the case that users without a medical exemption would be expected to wear the mask as a condition of flying.

B. In our setting, agents (aircraft passengers) commit to wear a mask, through the staking of a digital bond or token, in much the same manner that deposits are used to encourage recycling in certain countries. If a user complies with the social contract, all of the bond is returned, otherwise some of the bond is not returned.

D. Our system goes beyond that used in traditional circular economy applications in two ways. We use a distributed ledger (DLT) to record transfer of tokens from the users to the agency defining the social contract. This achieves two objectives. First, it allows the transfer of tokens in an automated and pseudo-anonymous manner. Second, it allows the implementation of feedback strategies to adjust the price of non-compliance in a personalised manner (i.e. allows the implementation of polluter-pays type models), while achieving a certain level of aggregate compliance.

E. As we shall see no information is recorded on the DLT other than an average level of compliance, and all information is private and pseudo-anonymised. Specifically, even though our mask uses Carbon-dioxide (eCO2) and Total Volatile Organic Compounds (TVOC) information to detect whether a mask is worn correctly, none of this, or any user specific biological information, is recorded on the DLT.

F. Finally, we emphasise that we are not proposing any type of licensing system. A user stakes a bond as a promise to fulfil a contract. This bond is returned when the contract is fulfilled. The functioning of this digital token system is similar to the process of using a shopping trolley in some supermarkets. To utilise the trolley, one needs to deposit a coin, and upon returning it, the coin is refunded. In our context, the token is only meant to be used as a deposit and incentive for individuals to follow social distancing protocols in specific situations, like a crowded airplane.

The remainder of this paper is organised as follows. In Section 2, we give a brief description of DLTs and more specifically of the IOTA distributed ledger, also known as the IOTA Tangle. In Section 3, we provide an overview of relevant work from both pricing algorithm and smart mask design perspectives. In Section 4, we present the Personalised Feedback Control Algorithm (PFCA) and the smart mask model designed in this paper. Section 5 validates the efficacy of the proposed approach through hardware-in-the-loop simulations and agent-based simulations. Section 6 summarises the results discussed in this work and outlines future research directions.

Finally, we note also that a short version of this paper has been accepted at the BLOCKCHAIN’23: 5th International Congress on Blockchain and Applications. A short summary of the results in this paper will be published in the conference proceedings (Springer).

## 2 Background

### 2.1 IOTA Tangle

The term *distributed ledger technology* (DLT) refers to digital database architectures that are shared across many nodes in a peer-to-peer network [13]. DLTs have recently gained popularity in both industry and in academia, and have found many applications. For example, they have been used in the context of smart city domains [10], in supply chain applications [14], and in the health-care domain [15]. DLTs hold great potential in these sectors because of several desirable properties such as, decentralization, immutability of records, and transaction transparency. Although Blockchain is the best known DLT architecture, several limitations hinder its widespread application in an IoT context [16]. For example, the inherent sequential structure in Blockchain to add new transactions often results in a limited transaction rate and the heavy cryptography based Proof-of-Work (PoW) consensus mechanism leads to expensive computation power.

As alternative to Blockchain based architectures is a ledger such as the IOTA Tangle. The structure of the IOTA Tangle is a Directed Acyclic Graph (DAG). In this DAG-based ledger, widely referred to as the Tangle, information is stored in a graph based structure rather than a sequential set of blocks. In the IOTA DAG, vertices of this graph are blocks issued by network participants, and each block contains at most one transaction. The edges of the graph are formed by blocks (transactions) approving previous blocks that we call *parents*. All yet unapproved transactions are called *tips* and the set of all unapproved blocks is called the *tips set*. A node selects tips to approve through a *tip selection algorithm*. To issue a block a node selects *k* blocks from the tip-set. This process, called *approval*, is represented by an edge in the graph. If there exists a directed edge from vertex *i* to *j*, we say *j* is *directly approved* by *i*. If there is a directed path from *i* to *j* we say that *j* is *indirectly approved* by *i*.

**Fig 2:**
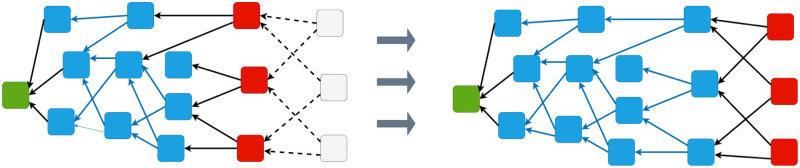
The Tangle structure.

As an example of this process, Fig 2 shows an instance of the Tangle with three new incoming blocks (left panel). Blue blocks have already been approved, red blocks represent the current tips of the Tangle and grey blocks are incoming transactions. Full details of the IOTA DAG can be found in [17, 18] (a description of Tangle version 1.4.3 can be found at https://iota.org/IOTA_Whitepaper.pdf).

## 3 Related work

We have already mentioned several related works in Section I. In particular, we mentioned that the notion of compliance arises in other application areas. In this section we also note that elements of related work can also be found in several other areas. For example, work based on pricing digital bonds are also related to other concepts in the literature [19, 20]. For example, our pricing algorithms are similar to work on dynamic pricing that can be found in several domains. One example of such work can be found in [21], where the author proposes a novel mechanism named User-Centric Dynamic Pricing (UCDP) to regulate the real-time urban traffic system. A novel dynamic toll-pricing algorithm is also proposed in [2] to alleviate traffic congestion. Similar concepts can be also found in the paper [3] which introduces a new smart parking system to allocate parking spaces. Other examples of related work in pricing can be found in [22, 23], where sellers to adopt flexible pricing policies according to different economic situations and customers’ specifications.

Other related work can be found in the area of smart mask design; especially in the context of COVID-19. [24] describes an active smart mask which is used for protecting people from coronaviruses and other airborne pathogens, such as pollutants or dust particles. A wearable smart mask integrated with a temperature sensor is described in [25] to continuously acquire human beings’ health data. To remotely measure temperature non-intrusively, especially to detect symptomatic people who might become infected with COVID-19 in public places, a smart mask prototype equipped with three thermistors is proposed in [26]. In [27], the author studied a smart mask prototype which integrates a reusable face mask shell and a sensing module that integrates with user-based mobile application. Although these works provide a solid foundation for our work, *none address the issue of personalised compliance with social contracts*.

There are also some works that approach the detection of mask-wearing, and enforcement of compliance with social contracts, through the use of Machine Learning (ML) techniques [28, 29]. For example, in [30], the author proposes an edge-computing-based mask (ECMask) identification framework, that uses facial recognition and deep learning methods to detect the mask wearing status. As another example in [31] the authors propose a system, also based on deep learning and computer vision technologies, that can achieve both mask face identification and temperature tracking. However, there are a number of differences between this approach and the one proposed in this paper, as well as limitations. Firstly, ML algorithms require a significant amount of data to build an accurate model. Secondly, in these approaches the authors do not make use of a pricing mechanism to regulate compliance with the social contract and they focus only on the detection of the mask status. Thirdly, most of the papers mentioned above require the use of face recognition methods, paired with ML algorithms to detect the status of the mask, whereas our proposals is based on hardware that detects locally the status of the mask, paired with the use of a token-based decentralised distributed ledger to protect the anonymity and the privacy of the users. Finally, in such situations, the ML technology simply detects non-compliance, and enforcement of mask wearing is the responsibility of observers, such as, for example, flight attendants, rather than the mask wearer (which is the main feature and advantage of our approach).

Other compliance related work appears in the context of game theory [7, 8]. However, there are also significant limitations within these works. For example, frequently such approaches are centralised; they are vulnerable to resource-rich attackers; they are often not anonymous because of unencrypted user addresses; and fairness is not addressed as they are not tailored according to the individual situation. All of these deficits can be addressed using the paradigm described in [4].

## 4 Mask model and algorithmic design

### 4.1 The PFCA algorithm

Before preceding to describe our mask design, we describe the motivation of employing PFCA algorithm. Although there are many works, such as game theoretic framework, privacy averse voting and recommendation platforms [7, 32], that aim to address the compliance issue, these strategies are vulnerable to manipulation by resource-abundant malicious actors, such as those making false recommendations. In contrast, the PFCA algorithm, which draws inspiration from stochastic approximations, hyperbolic discounting, and digital identity principles, adopts DLT and a series of control algorithms to enforce compliance without these drawbacks.

**Fig 3:**
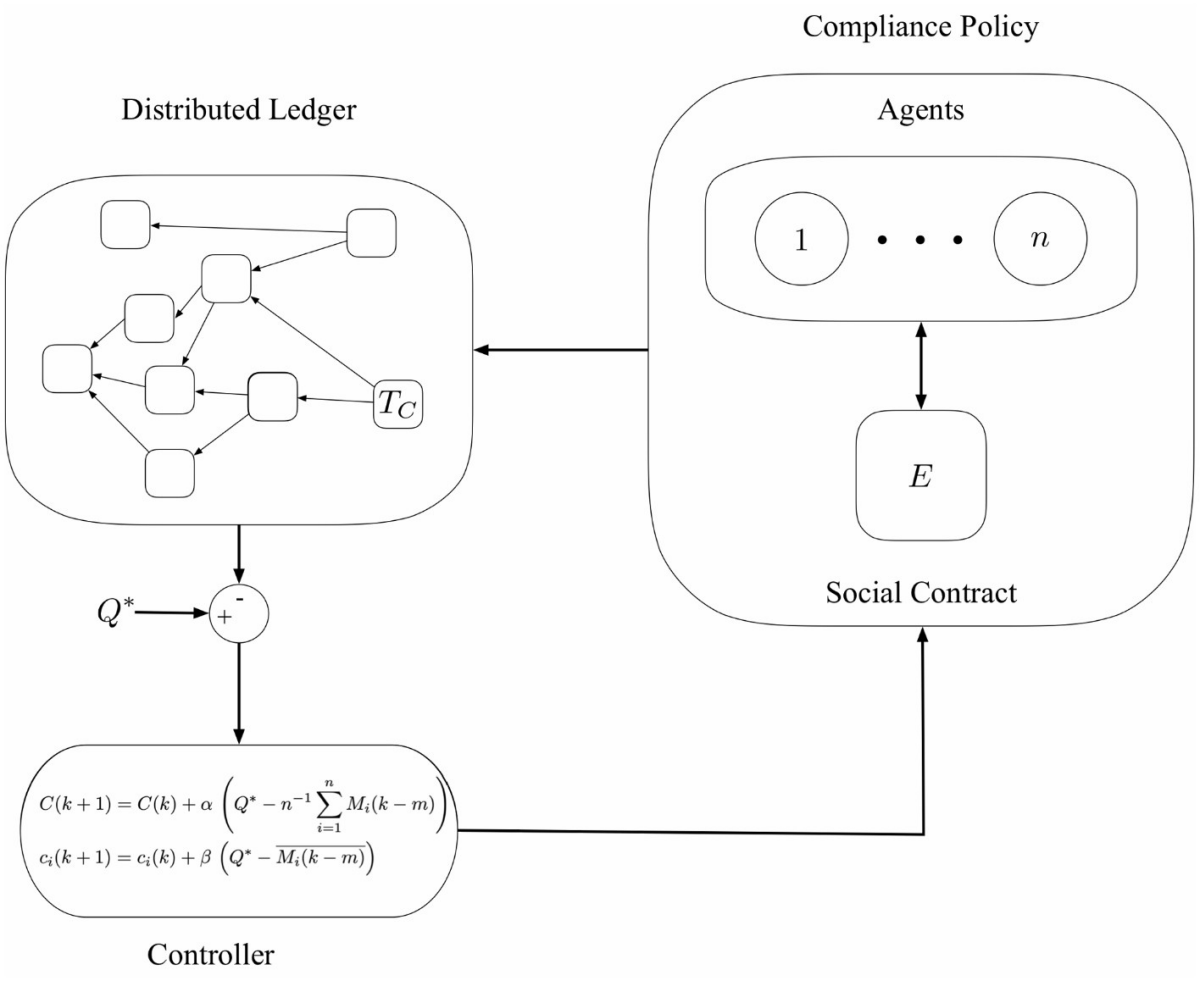
The functional components of the proposed compliance architecture are: The Distributed Ledger that acts as the communication backbone of the infrastructure, the compliance policy and the feedback controller [4].

Our work builds on [4], where digital tokens are enforce social contracts. A social contract defines guidelines that must be followed to ensure an agreed utilization of a shared object. The basic idea is that agents deposit tokens as a bond as an guarantee that they will utilise a resource or object in an agreed manner. If the object, such as a mask, is used in a correct manner (i.e. not removed), then the tokens are returned in full, or in part. An algorithm from [4] is used to determine the returned tokens based on level of compliance. The architecture described in [4] is shown in Fig 3. The system in Fig 3 operates as follows. A distributed ledger is used as a communication layer (i.e., to record the deposit and the withdrawal of the tokens) and a controller is used to adjust the number of tokens deposited and achieve expected compliance level for the social contract (compliance policy), denoted ***E***. Smart contracts mean that the entire system operates automatically. All operations are recorded on a DLT which is immutable and data are shared pseudo-anonymously among agents.

A feedback mechanism for designing a proper value of the bond is adopted from [4]. We consider *n* agents here and for each agent *i*, we define a binary random variable ${\left\{M_{i}\left(k\right)\in \left\{0,1\right\}\right\}}_{i=1}^{n}$ for discrete values *k* as:

$$\begin{array}{c} P\left(i\;\text{complies}\;\text{with}\;\text{rule}\;E\;\text{at}\;\text{time}\;k\right)=P(M_{i}\left(k\right)=1) \end{array}$$ (1)

This model essentially assumes that all agents behave rationally and adopts the approach to behavioural modelling described in [33] and in the follow-on papers [34–36]. This models essentially use iterated function systems to capture the concept of a rational agent. In what follows *q*_*i*_ is a constant parameter which represents the proclivity of each agent to comply with rules; *C*(*k*) and *c*_*i*_(*k*) represent, respectively, a global and an individual feedback signal whose purpose is to regulate the behaviour of each agent to achieve the desired level of compliance. The sum of *C*(*k*) and *c*_*i*_(*k*) represents the value of the token bond staked by agent *i* at time-step *k*; The combination *q*_*i*_ + *C*(*k*) + *c*_*i*_(*k*) is the probability that agent *i* will comply with the rule at time-step *k* + 1. Then, (1) can be expressed as

$$\begin{array}{c} P\left(M_{i}\left(k+1\right)=1\right)=p(q_{i}+C\left(k\right)+c_{i}\left(k\right)) \end{array}$$ (2)

with p:R→[0,1] being a monotone increasing function (which is used to bind the probability between 0 and 1). *C*(*k*) and *c*_*i*_(*k*) denote a global and an individual feedback signal respectively, which are designed to regulate each agent’s behaviour to reach the desired compliance level. However, since the agents employ a Distributed Ledger as their communication medium, due to the intrinsic delays that characterise these systems, their access is restricted to previous compliance levels represented as ${M_{i}\left(k-m\right)}_{i=1}^{n}$. In this notation, *m* denotes a delay in the measurements, a consequence of the PoW mechanism, ledger synchronisation, and time required for verification. Accordingly, we consider the following control laws, ∀k∈N and ∀*i* ∈ {1, …, *n*},

$$\begin{array}{c} C\left(k+1\right)=C\left(k\right)+\alpha (Q^{*}-n^{-1}\sum_{i=1}^{n}M_{i}\left(k-m\right) \end{array}$$ (3)

$$\begin{array}{c} c_{i}\left(k+1\right)=c_{i}\left(k\right)+\beta \;(Q^{*}-\bar{M_{i}\left(k-m\right)}) \end{array}$$ (4)

where *α* > 0 and *β* > 0 are two constants, ∀k∈N and ∀*i* ∈ {1, …, *n*}, Q* ∈ [0, 1] is the desired level of compliance, and $\bar{M_{i}\left(k\right)}$ is a windowed time average of the compliance of agent *i*, which is defined as

$$\begin{array}{c} \bar{M_{i}\left(k\right)}=\left(1-\gamma \right)\;\sum_{j=1}^{k}\gamma^{k-j}\;M_{i}\left(j\right) \end{array}$$ (5)

where (1 − *γ*)^−1^ is the length of the window for the average, with *γ* < 1.

The design of the algorithm is such that the value of the bond staked by agent *i* depends **not only** on the overall compliance of all agents, **but also** and how agent *i*’s past behaviour. The use of both a global and an individual control signal can be used to enforce *fairness* and resiliency from *pricing attacks*.

### 4.2 Mask design

**Fig 4:**
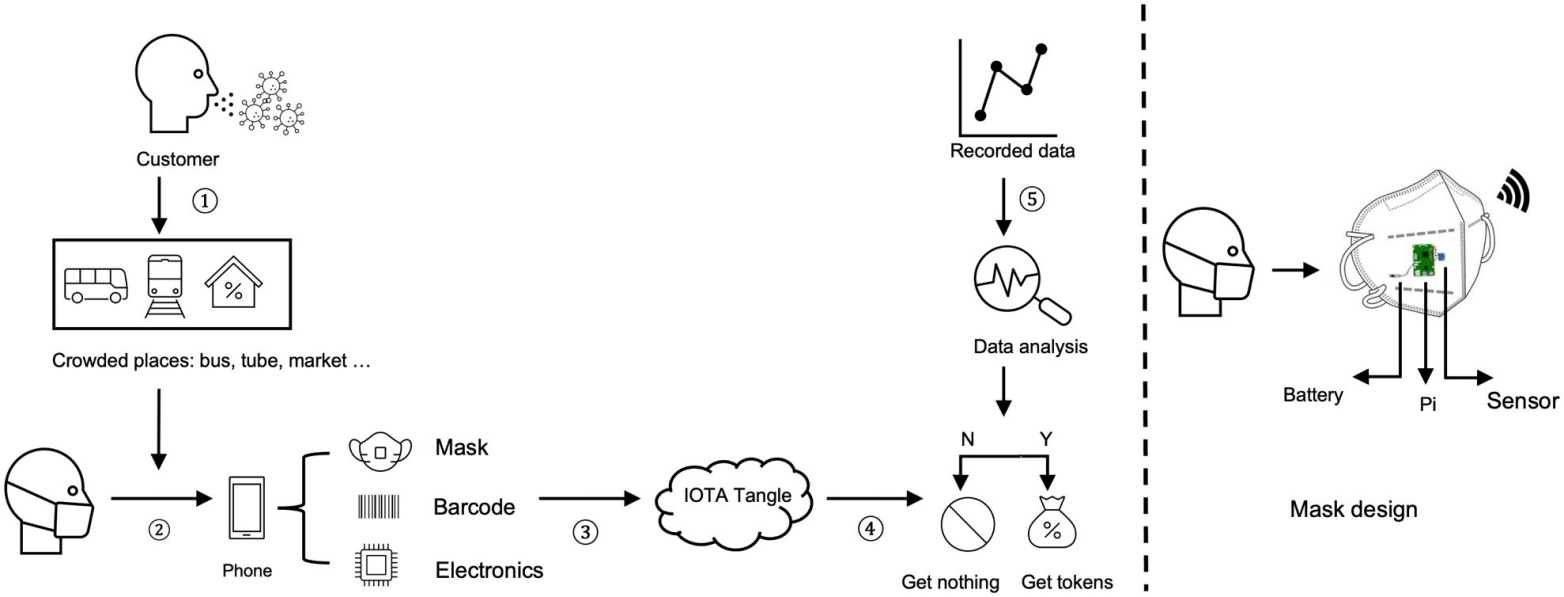
In the designed network structure, when customers enter into crowded places as shown in ①, such as bus, tube and markets, they are required to wear a mask paired with their phone as shown in ②. Each mask has a unique barcode and reusable electronics. During ③, the status of mask wearing is uploaded to IOTA Tangle. These recorded data are attached through ④ and analysed through ⑤. According to the compliance of the customer, the token bond is returned in full, or in part. In the right hand of the figure, we show the specific electronics for the system realisation which includes a battery, a raspberry Pi and a sensor.

Given the general background described above, we consider scenarios where agents move within a confined and/or crowded space where the use of masks is mandatory, such as buses, the tube, or airplanes. The following steps are performed to encourage social compliance and the structure of the network is depicted in Fig 4.

- Agents entering a space where mask wearing is mandatory must wear a smart mask that is connected to an agents phone. The mask is connected to a digital wallet via an integrated chip and the smart phone.
- An agent entering a restricted zone, deposits a number of tokens as a compliance bond. Smart contracts are used to automate this procedure.
- The sensor chip monitors Carbon-dioxide (eCO2) and Total Volatile Organic Compounds (TVOC), whereas the Raspberry Pi performs computations and uploads data to the IOTA Tangle in real time.
- If the agent wears the mask correctly, some, or all of its tokens are returned.

We now provide a brief description of the main hardware and software elements used for building the smart mask prototype.

- We used the IOTA *firefly digital wallet* for our mask design.
- A Raspberry Pi 3 is the main processing element to collect sensor data and to send and publish data to IOTA Tangle. The main task of the Pi is to implement the controller given in Eq (3). The controller could also be delegated to the smart-phone.
- Gas sensors, such as the, CJMCU-811 is used to monitor the density of eCO2 and TVOC exhaled by an agent.

Communication is based on Message Queuing Telemetry Transport (MQTT) [37] and Masked Authenticated Messaging (MAM) [15]. MQTT is a standard IoT protocol, and MAM is the IOTA communication protocol Both are very low-power consumption protocols.

In conclusion, from a practical standpoint, the smart mask system requires two components: (a) a smartphone, to perform computations and with an integrated digital wallet to communicate with the IOTA DLT; and (b) a smart-mask which consists of wearable-class electronics and a sensor.

**Fig 5:**
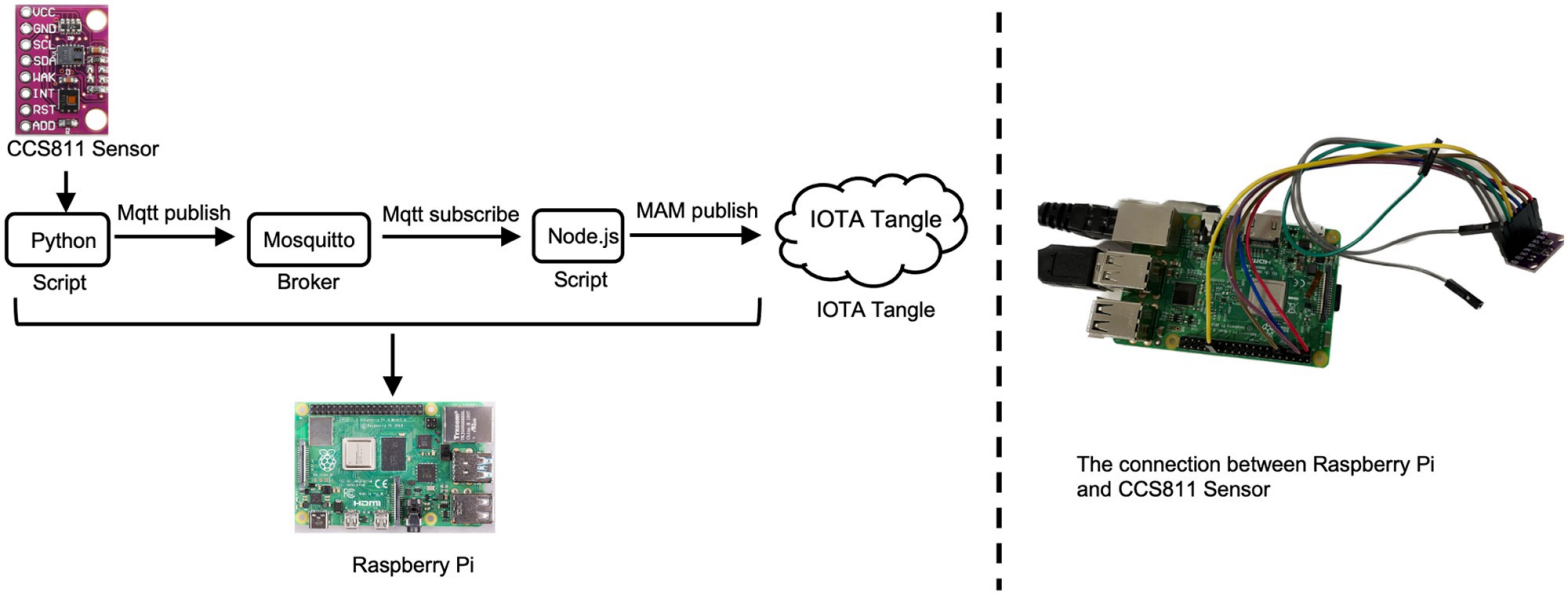
Implementation architecture including Raspberry Pi, CCS811 sensor and the communication protocol to IOTA Tangle.

Fig 5 shows that the network flow of uploading data to the IOTA Tangle. Agent-data is collected from the CJMCU-811 sensor which is connected to the Raspberry Pi 3B. The lightweight open source MQTT architecture is adopted to transfer data in the network. MQTT adopts a *publish and subscribe* model, in which some devices publish messages on a topic, while some devices which have subscribed to this topic receive this message. After the Raspberry Pi gathers eCo2 and TVOC data from the sensor, it then publishes these data to a specific MQTT topic. Other IoT devices such as the ESP32, ESP8266 boards, and other sensors, such as temperature and humidity sensors can also be added to the system monitor customers’ status. The module (depicted in Fig 5) named Node.js is used as the back-end server to subscribe to this specific topic and publish contained data in this topic to the IOTA Tangle through a lightweight data transmission protocol-MAM (see below).

MAM is a lightweight data transmission protocol designed by IOTA foundation. It enables nodes (any IoT device that can send or propagate transactions) to share encrypted data streams to each other node over the IOTA Tangle [38]. MAM is an important method for securing the transfer or accessing of the data stored in the IOTA Tangle. Nodes or devices, which are connected to the IOTA Tangle acting as publishers, broadcast their encrypted messages into a specified channel, while nodes that are interested in receiving the published messages can subscribe to the same channel [15]. There are three modes within MAM, including a Public mode, a Private mode, and a Restricted mode. For a public MAM mode, it employs the address of the transaction which is the same as Merkle Tree’s root [39]. The private MAM mode employs the addresses of transactions which is gained by hashing the root of the Merkle Tree, which means the message can be known only if the root of the Merkle Tree is obtained. For the restricted MAM mode, it employs the address of the transaction which is obtained by the hash of the Merkle tree’s root and a side key, which means the message can be known only if both the key and the root are known [40].

## 5 Experimental set-up

To illustrate the effectiveness of the designed algorithm, we set up two different types of simulations. The code can be found in https://github.com/Mona566/Mask-Paper.git.

- *Agent-based simulation*: An agent-based simulation is employed to mimic the behaviour of multiple agents within a room. Each agent behaves according to the equations described in Section IV. This simulation shows that with the proposed control strategy, the desired compliance is achieved and the probability of people being infected is reduced.
- *Hardware-in-the-loop simulation*: A user wears the mask prototype and its position and mask wearing status are respectively recorded to the IOTA Tangle and sent to the agent-based simulator in real time, as if the user was one of the agents of the simulations. This simulation is used to give real agents (people) the feeling of using a smart mask in a crowed area and to show that the interaction of the hardware and the control strategy works seamlessly.

In what follows, we provide a detailed description of each of these.

### 5.1 Agent-based simulation

We make use of an agent-based model to simulate the spread of COVID-19 within a confined space. In this experiment, we assume the number of agents is 500. Whenever agent *i* is in close range of agent *j*, i.e. when ||*x*_*i*_ − *x*_*j*_||_2_ ≤ *ϵ*, there is a probability that agent *i* will become infected. This probability is defined as

$$\begin{array}{c} P\left(i\;\text{becomes}\;\text{infected}\right)=P_{0}\left(1-m_{i}M_{i}\left(k\right)\right)\left(1-m_{j}M_{j}\left(k\right)\right) \end{array}$$ (6)

where the base infection rate is denoted by *P*_0_ (the chance of being infected with no social distancing and mask wearing), *m*_*i*_ ∈ [0, 1] is the effectiveness of the mask worn by agent *i*, and *M*_*i*_(*k*) ∈ {0, 1} is a binary random variable that indicates whether agent *i* is wearing a mask at time instant *k*.

**Fig 6:**
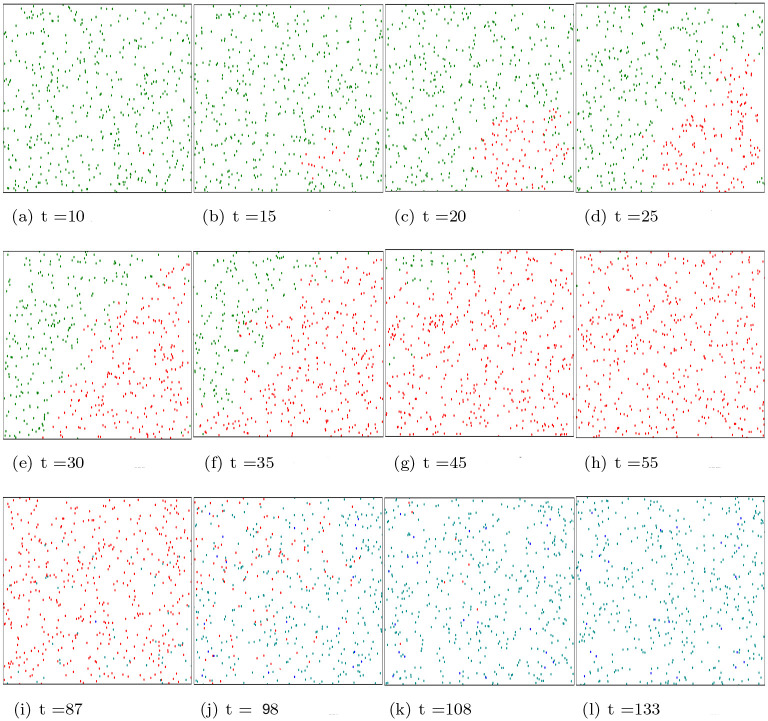
The degree of infection among people when they do not wear masks properly at different time instants. Green dots denote healthy people, red dots denote infected people, cyan dots denote immune people with slight possible sequelae, while blue dots denote immune people with serious sequelae.

**Fig 7:**
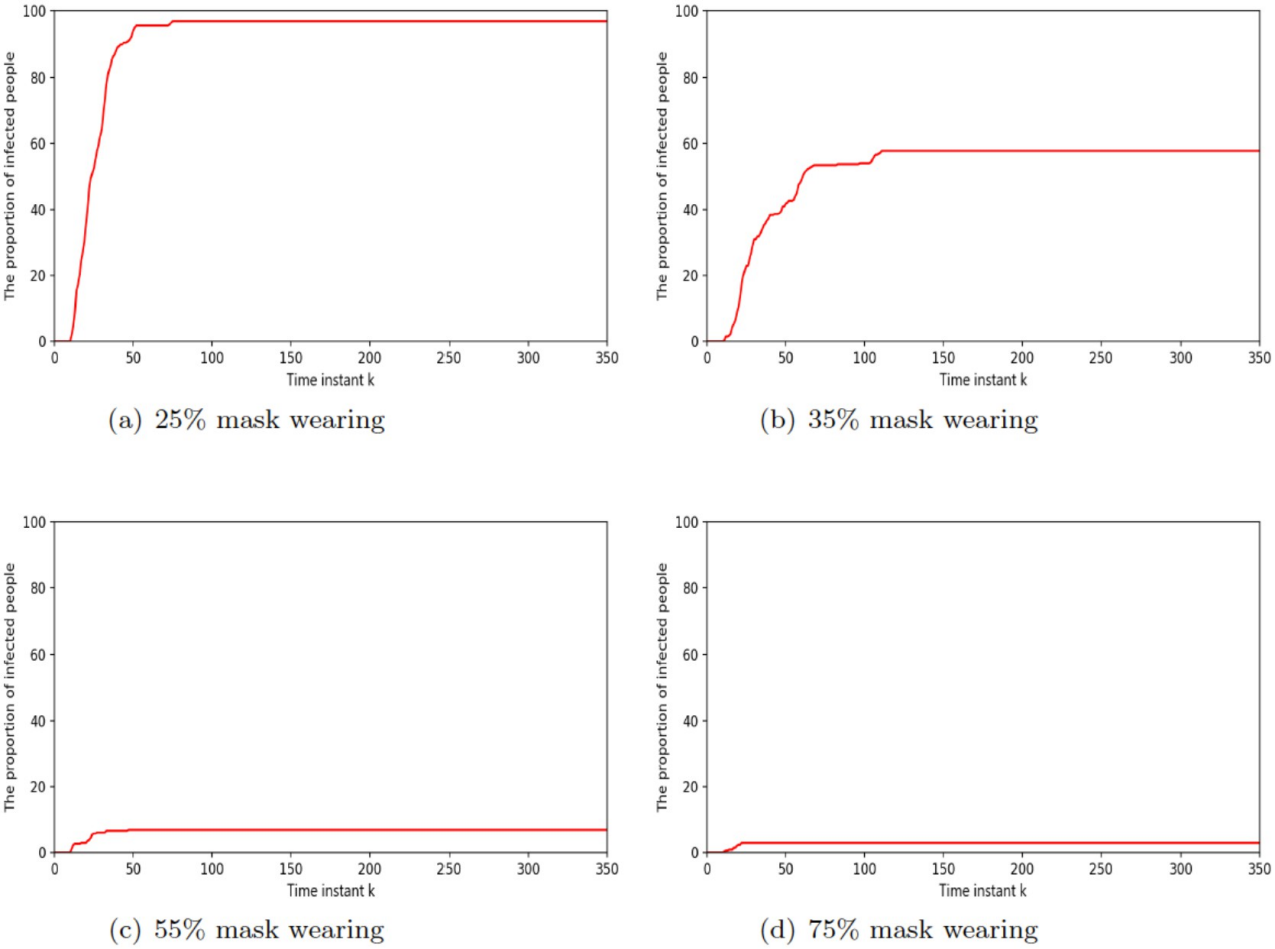
The proportion of infected people, as a time series, in different mask wearing scenarios.

**Fig 8:**
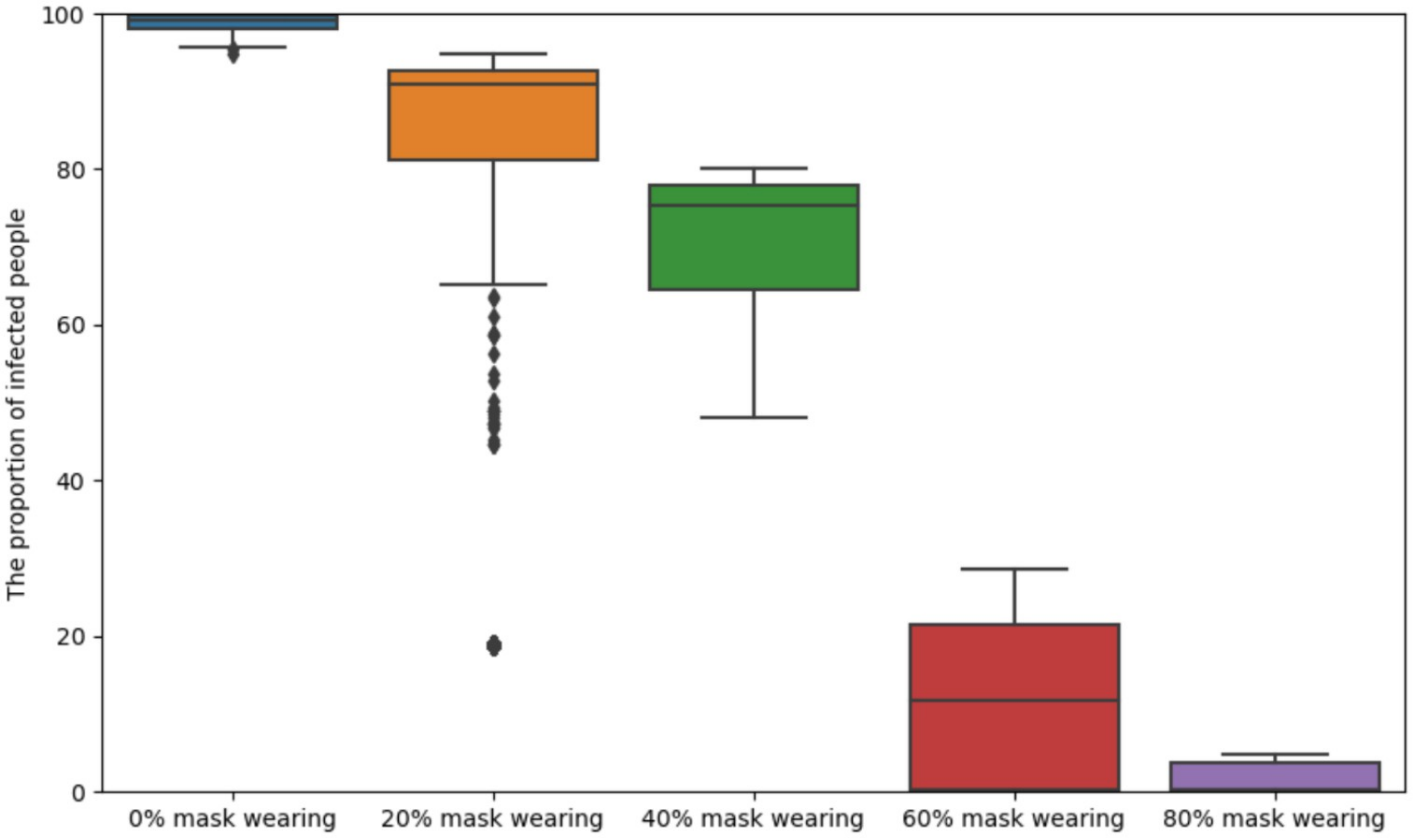
Boxplots corresponding to Fig 7. Boxplot results are produced from 25 Monte Carlo simulations, each running 250 time units. When there is no compliance with mask wearing (0%), the infection rate is exceptionally high. The median proportion of infected individuals is approximately 90% under the scenario of 20% mask-wearing compliance, and median proportion of infected individuals is approximately 75% when the compliance with mask wearing increases to 40%. Notably, as mask-wearing compliance rises to 70%, there is a substantial reduction in the median infection rate to around 10%. Furthermore, when mask-wearing compliance reaches 80%, the maximum infection rate significantly decreases to approximately 5%.

- When no masks are worn the infection rate increases very quickly. As shown in Fig 6(a), we set up the model with a single infected individual, marked as the red dot. As depicted in Fig 6(b)–6(h) the virus spreads very quickly. At *t* = 30, as depicted in Fig 6(e), nearly half of people become infected and nearly all agents become infected from *t* = 55 as depicted in Fig 6(h).
- When agents, including healthy agents and infected agents wear masks effectively, the infection rate and serious infection rate increase at a slower rate. As depicted in Fig 7(a), when 25% of agents wear masks, the proportion of infected agents is almost 100% around 50 time instant. As depicted in Fig 7(b), when 35% of agents wear masks, the maximum percentage agents that become infected is also around 60%. As depicted in Fig 7(c), when 55% of agents are wearing masks, the maximum percentage of infected agents is only around 10% at 50 time instant. Finally, Fig 7(d) shows that the amount of infected agents is even lower if 75% people are wearing masks. Fig 8 displays the error bar for mask-wearing performance. The corresponding boxplot results are produced from 25 Monte Carlo simulations, each running 250 time units. When there is no compliance with mask wearing (0%), the infection rate is exceptionally high. The median proportion of infected individuals is approximately 90% under the scenario of 20% mask-wearing compliance, and median proportion of infected individuals is approximately 75% when the compliance with mask wearing increases to 40%. Notably, as mask-wearing compliance rises to 70%, there is a substantial reduction in the median infection rate to around 10%. Furthermore, when mask-wearing compliance reaches 80%, the maximum infection rate significantly decreases to approximately 5%.

**Fig 9:**
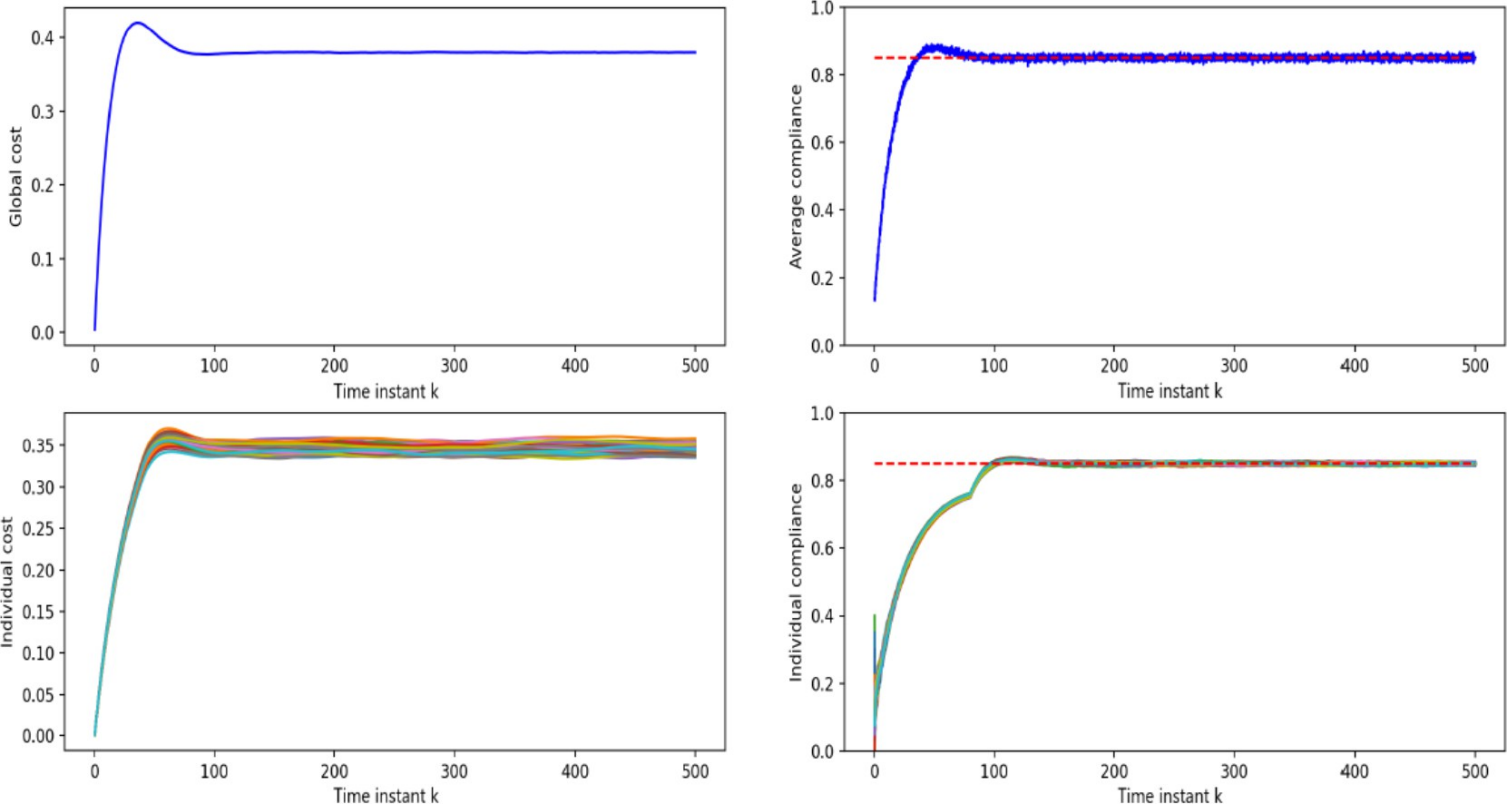
Global cost and individual cost. The above plot is global cost which is calculated by Eq (3); The bottom plot is individual cost which is calculated by Eq (4); (b) Global compliance and individual compliance.

These simulations show, qualitatively, that high levels of compliance with social distancing norms lead to lower infection rates overall. This is the rationale behind the employment of the control laws described in Section II. In fact, as depicted in Fig 9, with PFCA including both a global and an individual cost signal, the desired level of compliance is achieved and the fairness of cost among all individuals is also ensured (as each individual will comply equally, regarding of their initial proclivity *q*_*i*_).

**Remark**: We want to emphasise that, the agent model used in this paper does not intend to be a realistic simulation of the dynamics of how COVID-19 spreads across a given population. Rather it is intended as a elementary model, to showcase how the control algorithm and the smart mask would work in a scenarios similar to a viral spread in a population of agents.

### 5.2 Hardware-in-the-loop simulations

**Fig 10:**
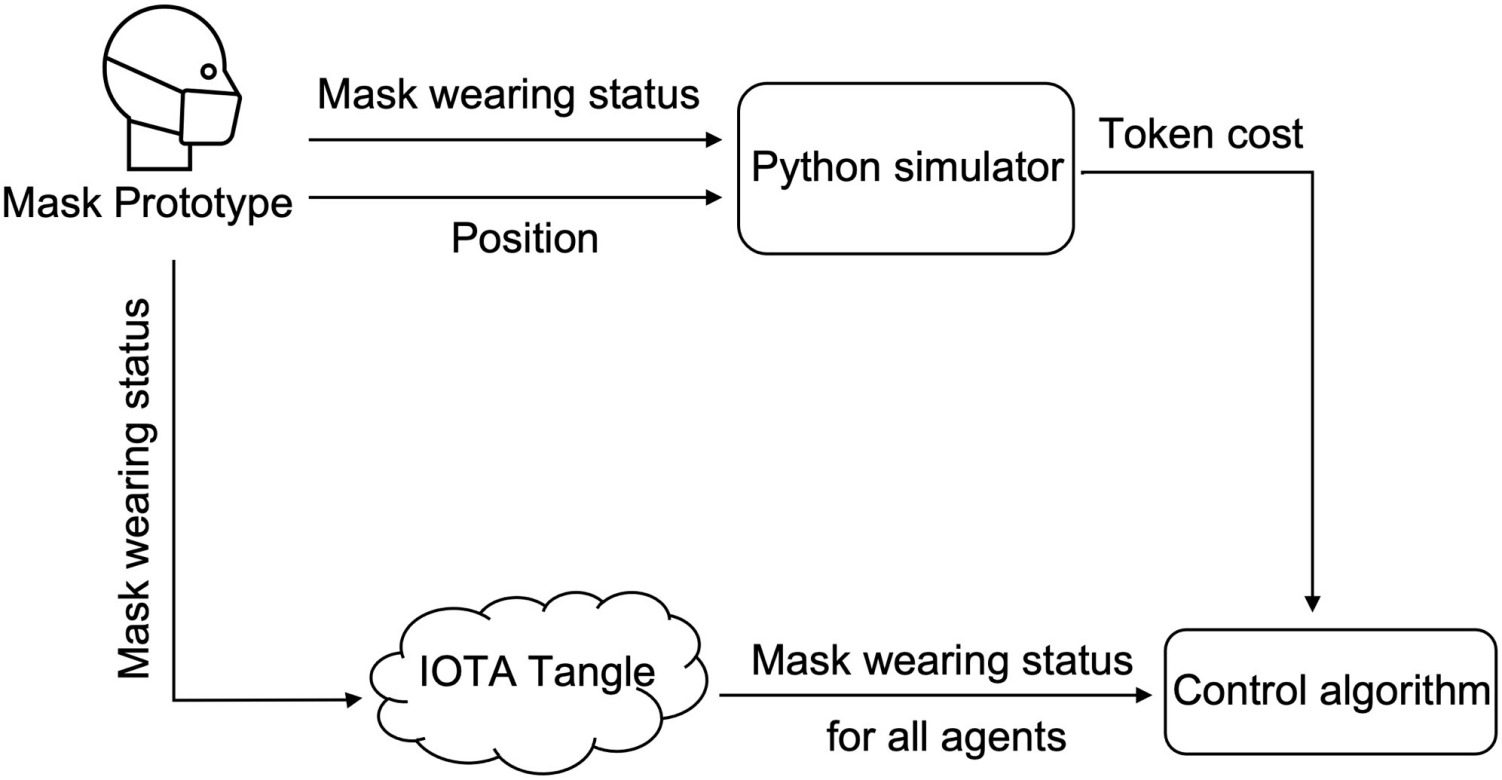
The structure of hardware-in-the-loop simulation.

**Fig 11:**
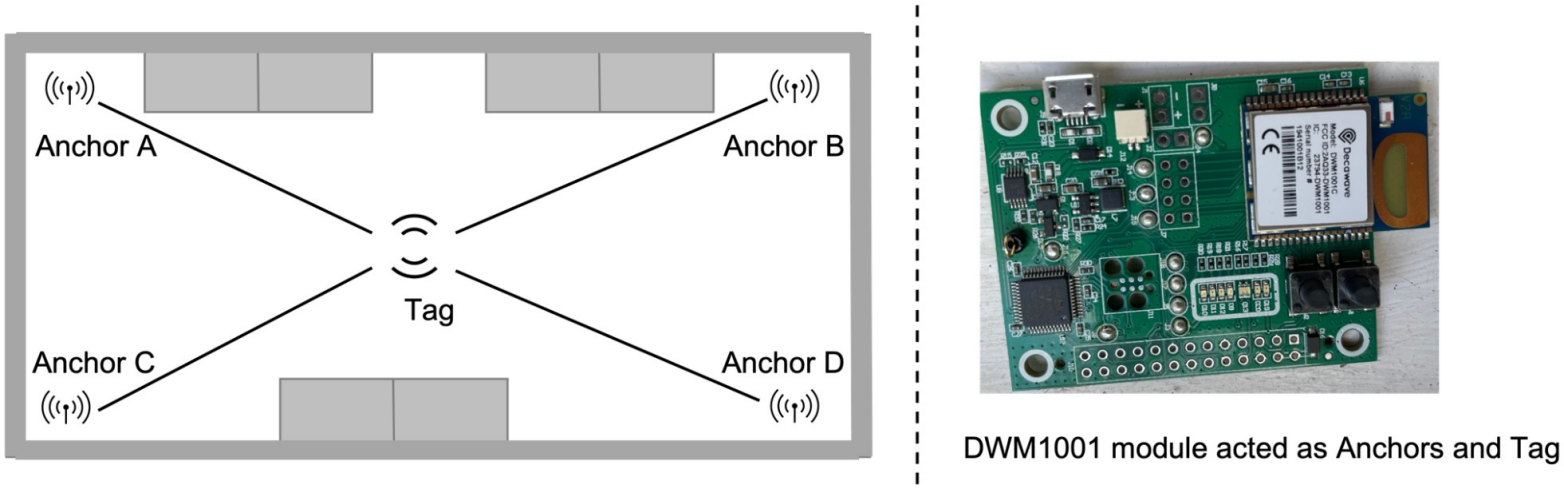
Experimental setup for indoor positioning system.

**Fig 12:**
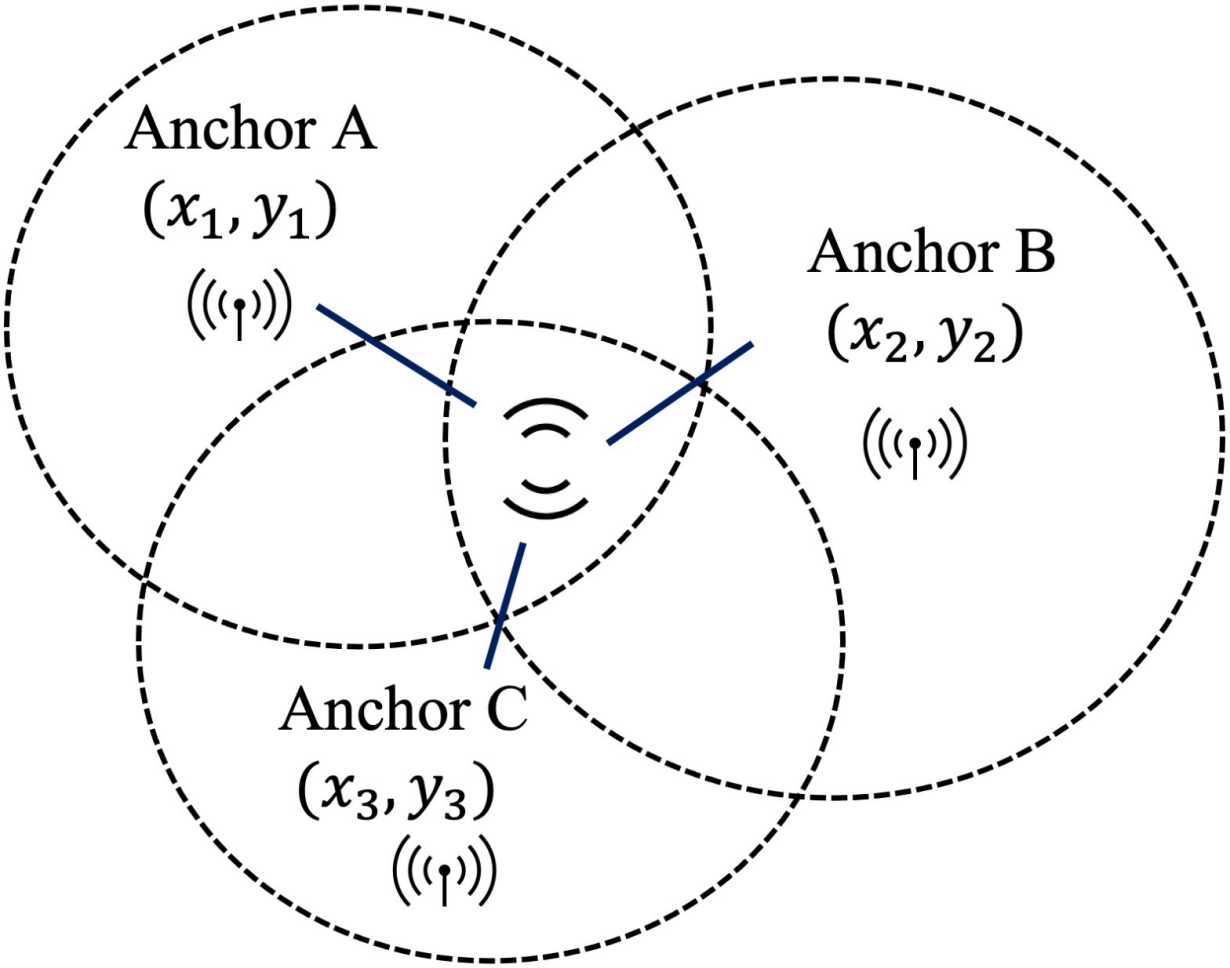
Utilizing ToF triangulation to perform object localization.

In the previous section, we showed that with the proposed control strategy, the desired compliance is achieved and the probability of people being infected is reduced. In order to give real agents (people) the feeling of using a smart mask in a crowed area, we develop a hardware-in-the-loop simulation based on indoor positioning. As shown in Fig 10, the hardware-in-the-loop simulation is used for monitoring the smart mask wearing position and status of a real agent. The position and the status of the mask are both sent to the IOTA Tangle and the python simulation in which a number of virtual agents are emulated, and all data for both real and virtual agents is sent to the IOTA Tangle. Detection of the mask position is based on Ultra-Wide Band (UWB) indoor positioning technology [41]. The equipment used in these experiments is the DW1001-DEV. We use the Decawave concepts of ‘Anchor’ and ‘Tag’ to denote different UWB nodes in the system. An ‘Anchor’ represents a fixed node whose position is known. Anchors use wireless signals from tags to determine the position of movable tags whose position is unknown. In this experiment, the architecture for indoor positioning is depicted in Fig 11. We set four DWM1001 modules as Anchors and one DWM1001 module as a tag (representing the customer moving within a closed space). Embedded firmware provides two-way ranging (TWR) and real time location system (RTLS) functionality are pre-loaded in the DWM1001 module; see https://www.Decawave.com. Note that Decawave adopts the TWR method which is based on time of flight (ToF) [42] to obtain range measurements. In addition, as shown in Fig 12, the triangulation technique is employed to determine the tag’s position within a 2-D environment that has three or more anchor points [43]. The distance measurement behind this method is that it takes the product of a measured time and the speed of light. The measured time is the radio signal that travels between an emitter and a receiver. The principle behind To measure distance, three messages named *Poll, Response* and *Final* need to be exchanged between anchors and tag. Anchor timestamps and tag timestamps are recorded and used to calculate the distance *D*_*i*_*s*.

**Fig 13:**
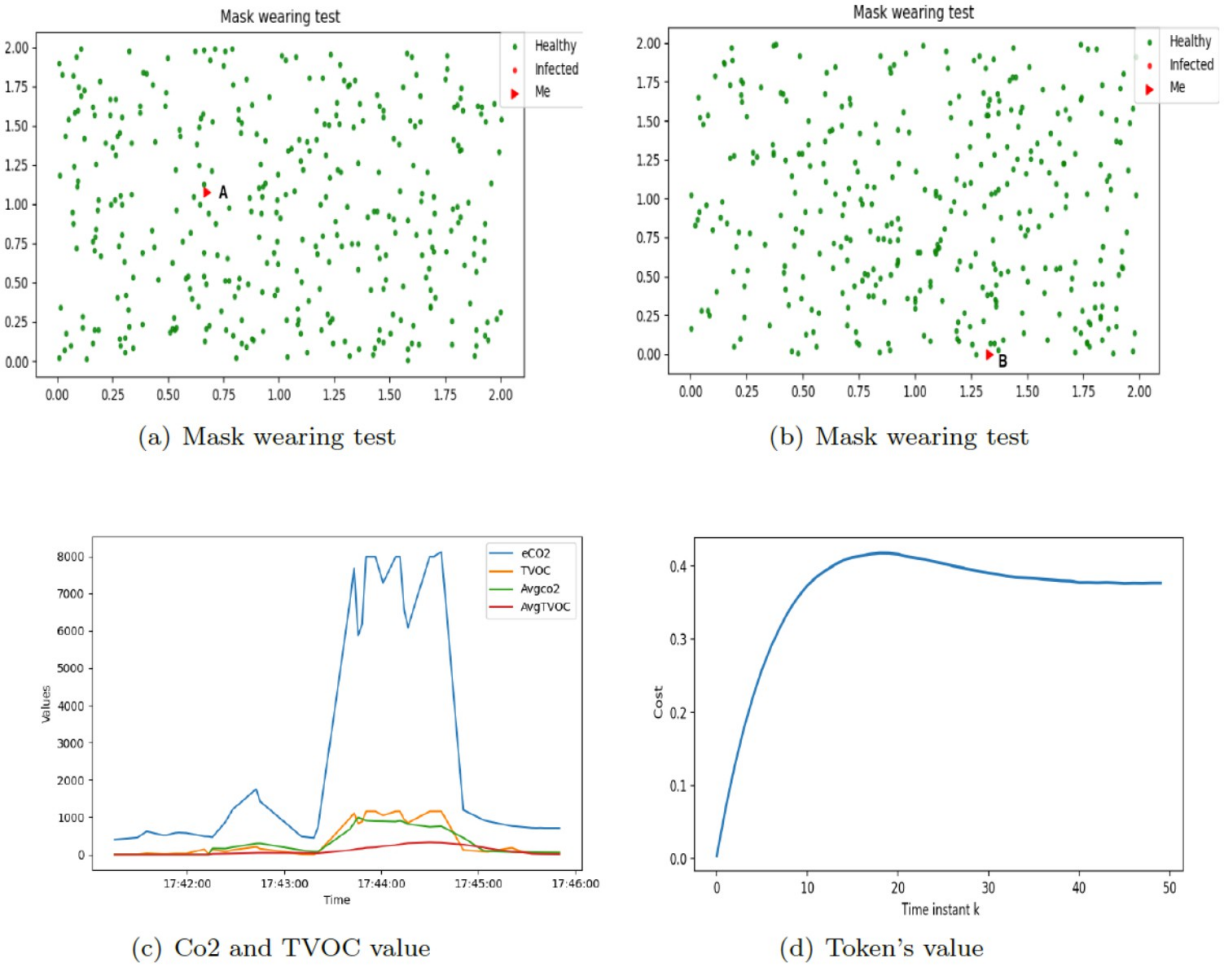
Mask wearing test. (a) The red triangle depicts the position of the Tag, which is at point A. (b) The red triangle depicts the position of the Tag, which is at point B. Red and green dots denote other agents generated by the simulator, in which green dots present healthy agents and red dots denote infected agents. (C) The level of *eCo2*, *TVOC*, *AvgCo2* and *AvgTVOC*. We use blue line for *eCo2*, red line for *TVOC*, yellow line for *AvgCo2* and green line for *AvgTVOC*. (d) The variation of the individual cost.

Experiments were conducted in a room at Imperial College London. There are four anchors named DW4105, DW9B10, DW4A2F (as initiator), DW4C15, and one Tag named DW181C. In Fig 13(a) and 13(b), the triangle depicts the position of the Tag (i.e., the PoC-based mask), while other dots denote other agents generated by the simulator. Fig 13(c) depicts the data collected by the gas sensor. The processing of the data is done as follows.

- In order to account for the noise from sensors we average the data, effectively applying a low pass filter to them, over a fixed window of time. For these experiments the time window was fixed at 10 samples.
- In order to detect whether the mask was worn or not, we set two thresholds for eCo2 and TVOC. If the computed moving averages are above these thresholds we know that the mask was worn correctly. The two thresholds are set, respectively, at 500 ppm for eCo2 and 50 ppb for TVOC.

**Remark**: This experiment that we have described is designed to illustrate the main features of the operation of the system. In particular, the eCO2 and TVOC thresholds are chosen empirically. Any practical implementation of the system would require these factors to be selected in a more rigorous manner by considering factors such as the mask wearers’ age, health, and environmental conditions (e.g., air pressure).

As mentioned above, Fig 13(c) shows the values for Co2 and for TVOC, as detected from the mask. It is possible to appreciate, even by visual inspection, that the reading from the sensors increase drastically when somebody is wearing a mask, making it straightforward to detect, whether or not a user is complying. Finally, Fig 13(d) depicts the variation of individual cost, paid by the specific user according to the compliance.

## 6 Conclusion

In this paper, a smart mask prototype is designed to monitor people’s mask wearing status. The use of DLT-based IOTA Tangle, serves as both a communication layer for the control algorithm as well as ledger ensuring the security and immutability of data. The controller is used to form a feedback loop to regulate the network. The designed mechanism is validated through extensive simulations including a python-based one and a hardware-in-the-loop one. Planned future work includes evaluation of the mask in real urban settings, to develop the compliance policies developed in [3], and to evaluate user acceptance in population studies for the compliance based systems, all of which constitute limitations of the present work.
